# *Boswellia serrata* Extract and Its Bioactive Compound 3-O-Acetyl-11-Keto-β-Boswellic Acid (AKBA) Induce ROS-Mediated Intracellular Clearance of *Porphyromonas gingivalis* in Human Gingival Epithelial Cells

**DOI:** 10.3390/ijms27041733

**Published:** 2026-02-11

**Authors:** David Vang, Pedro Henrique Carneiro, Laura Henao, Adrien Stroumza, Harmony Matshik Dakafay, Scott Davis, David M. Ojcius, Cassio Luiz Coutinho Almeida-da-Silva, Aline Cristina Abreu Moreira-Souza

**Affiliations:** 1 Department of Biomedical Sciences, Arthur A. Dugoni School of Dentistry, University of the Pacific, San Francisco, CA 94103, USApnascimentocarneirodasilva@pacific.edu (P.H.C.); laurahenao205@gmail.com (L.H.); dojcius@pacific.edu (D.M.O.); 2 Dental Surgery Program, Arthur A. Dugoni School of Dentistry, University of the Pacific, San Francisco, CA 94103, USA

**Keywords:** *Boswellia serrata*, AKBA, *Porphyromonas gingivalis*, reactive oxygen species, ROS, natural products, periodontitis

## Abstract

*Porphyromonas gingivalis* is a keystone pathogen in periodontitis, known for its ability to invade gingival epithelial cells and persist intracellularly. Conventional antimicrobials are often ineffective against intracellular pathogens, and natural products remain poorly explored in this context. Here, we investigated the antimicrobial effects of *Boswellia serrata* extract and its bioactive compounds on the dynamics of *P. gingivalis* infection in human gingival epithelial cells. During early times of infection, *B. serrata* extracts stimulated phagocytosis and increased bacterial internalization, suggesting modulation of epithelial uptake mechanisms. At later times of infection, *B. serrata* increased production of reactive oxygen species (ROS) in host cells and markedly reduced intracellular bacterial load. The antimicrobial effect was abolished by the ROS scavenger N-acetylcysteine, confirming a role for oxidative mechanisms in the clearance of *P. gingivalis*. Similar results were obtained with 3-O-acetyl-11-keto-β-boswellic acid (AKBA), one of the major boswellic acid derivatives found in *B. serrata* extract. These findings reveal a dual role of *B. serrata* compounds in response to *P. gingivalis* infection, in which *B. serrata* initially facilitates bacterial entry and subsequently promotes ROS-dependent intracellula These findings provide new mechanistic insights into the regulation of host–pathogen interactions by the natural products found in *B. serrata*. Our results support the therapeutic potential of *B. serrata*-derived compounds for managing periodontal infections.

## 1. Introduction

*Porphyromonas gingivalis* is a key intracellular pathogen in periodontitis, capable of invading human gingival epithelial cells and persisting within tissues, thus contributing to the chronic inflammatory characteristic of the disease [1,2,3,4]. Intracellular persistence of *P. gingivalis* is facilitated by its ability to evade host defenses and modulate local host immune responses [1,5,6,7,8,9], which contribute to *P. gingivalis*’ association with oral and several systemic diseases [4]. The increasing rate of antibiotic resistance and the fact that conventional antibiotics may not always be successful against intracellular pathogens highlight the need for alternative strategies to eliminate pathogens [10]. Natural products are considered safer for human consumption than synthetic drugs and, therefore, represent ideal candidates for therapeutic treatments [11].

Frankincense oil can be extracted from *Boswellia serrata*, a medicinal plant widely recognized for its anti-inflammatory properties [11]. The *B. serrata* extract contains bioactive boswellic acids with diverse biological activities, including antimicrobial effects against oral pathogens [11,12,13]. We have previously reported that the *B. serrata* extract eliminates *P. gingivalis* planktonic cell growth and reduces *P. gingivalis* biofilm formation [13]. Other groups have shown that different compounds from *B. serrata* inhibit other oral pathogens, such as *Streptococcus mutans* and *Enterococcus faecalis* [12]. Boswellic acids and several of their acetylated components exhibit antibacterial activity against Gram-positive bacteria and can synergize with antibiotics, making them potential therapeutic agents [14].

Reactive oxygen species (ROS)-mediated killing is a canonical host defense mechanism employed by immune cells and epithelial cells [15,16,17,18]. ROS production plays a central role in epithelial antimicrobial activity, directly damaging intracellular pathogens and promoting their clearance [19]. Boswellic acid compounds have been shown to induce ROS production in host cells [20,21,22,23]. Mechanistic studies demonstrate that 11-keto-boswellic acids stimulate ROS synthesis in polymorphonuclear leukocytes via NADPH oxidase and MAPK signaling in a Ca^2+^-dependent manner [24]. However, *B. serrata* and related boswellic acids can also exhibit antioxidant effects in non-immune cells, indicating that responses may vary depending on the cell type [13].

Our recent studies demonstrate that *B. serrata* extracts inhibit the growth of *P. gingivalis* and biofilm formation, and can reduce intracellular infection in human gingival epithelial cells, without exhibiting cytotoxic effects [13]. These findings suggest that bioactive *B. serrata* compounds may act by modulating host responses to limit intracellular survival of *P. gingivalis*, although the precise mechanisms underlying this effect remain to be elucidated.

We hypothesize that bioactive compounds from *B. serrata* restrict *P. gingivalis* infection in human gingival epithelial cells by enhancing host antimicrobial responses. Here, we demonstrate that treatment with *B. serrata* compounds reduced intracellular bacterial load and increased ROS production, while ROS scavenging reversed the antimicrobial effect. Understanding the cellular mechanism and identifying the bioactive compounds from *B. serrata* involved in this effect may support the development of strategies for managing periodontal infections.

## 2. Results

### 2.1. B. serrata Enhances P. gingivalis Internalization and Epithelial Phagocytic Activity During Early Times of Infection

**Figure 1:**
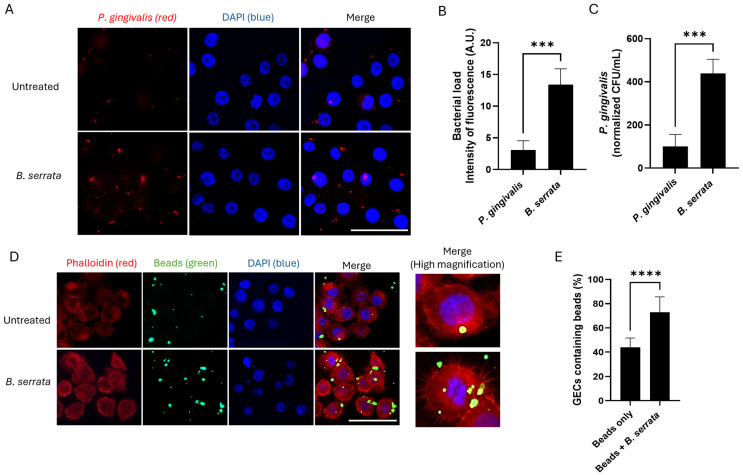
*B. serrata* enhances phagocytosis of *P. gingivalis* by human gingival epithelial cells during early times of infection. (**A**) Immunofluorescence micrograph showing intracellular *P. gingivalis* (red) infection in human gingival epithelial cells incubated with or without *B. serrata* extracts, at 5 h post-infection (scale bar = 50 µm). (**B**) Quantification of intracellular fluorescence intensity in *P. gingivalis*-infected cells incubated with or without *B. serrata* extracts, at 5 h post-infection (*** = *p* < 0.001, n = 3). (**C**) CFU quantification of viable *P. gingivalis* incubated with or without *B. serrata* extracts, at 5 h post-infection (*** = *p* < 0.001, n = 4). (**D**) Representative fluorescence micrograph of human gingival epithelial cells incubated with green-fluorescent beads in the presence or absence of *B. serrata* extract at 5 h post-infection (scale bar = 50 µm). (**E**) Percentage of bead-containing gingival epithelial cells at 5 h post-infection (**** = *p* < 0.0001, n = 4).

To assess the effects of *B. serrata* extracts on early times of *P. gingivalis* infection, human gingival epithelial cells were incubated with *B. serrata* extracts and infected with *P. gingivalis* simultaneously for 2 h. Our immunofluorescence experiments revealed increased bacterial infection in *B. serrata*-treated cells compared with untreated controls, at 5 h post-infection (Figure 1A). Quantification of fluorescence intensity for *P. gingivalis* (red) (Figure 1B) and colony-forming units (Figure 1C) confirmed a significant increase in bacterial internalization. To determine whether this effect reflected an increase in bacterial invasion ability or an increase in phagocytic activity, we performed our infection protocol using inert green-fluorescent microspheres (beads sizing 0.5 or 1 µm), instead of *P. gingivalis*, and stained the actin with phalloidin (red). Our fluorescence micrograph (Figure 1D) and corresponding quantification (Figure 1E) show an increased prevalence of bead-containing gingival epithelial cells under *B. serrata* treatment compared to untreated controls. The inserts also highlight cytoplasmic extensions present only in *B. serrata*-treated cells. These findings suggest that *B. serrata* facilitates phagocytosis of *P. gingivalis* during early times of infection.

### 2.2. B. serrata Limits P. gingivalis Persistence at Later Times of Infection

**Figure 2:**
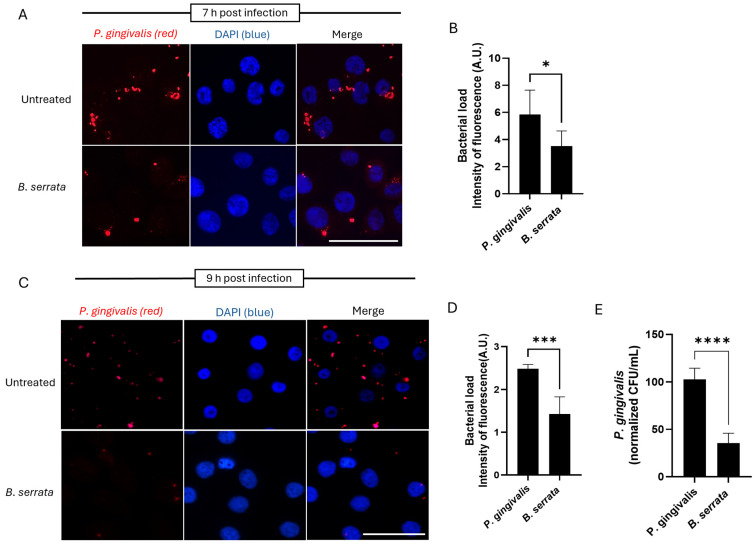
*B. serrata* reduces the intracellular load of *P. gingivalis* in human gingival epithelial cells. (**A**) Immunofluorescence micrograph showing intracellular *P. gingivalis* at 7 h post-infection in untreated control and *B. serrata*-treated gingival epithelial cells (scale bar = 50 µm). (**B**) Quantification of intracellular intensity of fluorescence in *P. gingivalis*-infected cells incubated with or without *B. serrata* extracts, at 7 h post-infection (* = *p* < 0.05, n = 3). (**C**) Immunofluorescence micrograph showing intracellular *P. gingivalis* at 9 h post-infection in untreated control and *B. serrata*-treated gingival epithelial cells (scale bar = 50 µm). (**D**) Quantification of intracellular intensity of fluorescence in *P. gingivalis*-infected cells incubated with or without *B. serrata* extracts, at 9 h post-infection (*** = *p* < 0.001, n = 3). (**E**) CFU quantification of viable intracellular *P. gingivalis* in the presence or absence of *B. serrata* treatment at 9 h post-infection (**** = *p* <0.0001, n = 4).

To determine whether *B. serrata* affects the intracellular persistence of *P. gingivalis* at later times of infection, human gingival epithelial cells were infected with *P. gingivalis* in the presence or absence of *B. serrata* extracts, and the bacterial load was analyzed at 7 h and 9 h post-infection. Our immunofluorescence micrograph (Figure 2A) and corresponding fluorescence quantification (Figure 2B) revealed a marked reduction in intracellular bacteria at 7 h post-infection in *B. serrata*-treated cells compared with controls. Similarly, we detected a decrease in bacterial load (Figure 2C,D) at 9 h post-infection in *B. serrata*-treated cells compared with untreated controls. Our CFU quantification experiments at 9 h post-infection demonstrate a significant reduction in metabolically active intracellular *P. gingivalis* in human oral cells treated with *B. serrata* during infection (Figure 2E), which supports our immunofluorescence microscopy data in Figure 2C,D. These findings suggest a dual role for *B. serrata*-stimulated host responses against infection, in which *B. serrata* initially promotes uptake of bacteria but enhances bacterial clearance at later times of infection.

### 2.3. B. serrata Induces ROS Production and Restricts Intracellular P. gingivalis Survival in Human Gingival Epithelial Cells

**Figure 3:**
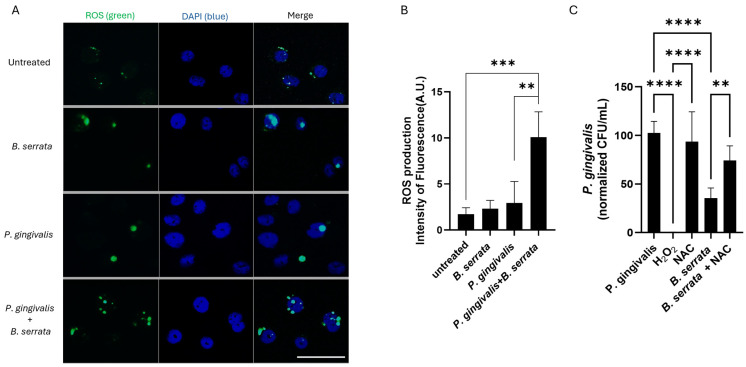
*B. serrata* induces ROS production and restricts the intracellular survival of *P. gingivalis* in human gingival epithelial cells. (**A**) Representative fluorescence micrograph of ROS generation in human gingival epithelial cells infected or not with *P. gingivalis* and treated with or without *B. serrata* extracts. Detection of ROS using the CellROX fluorogenic probe was performed 7 h post-infection (scale bar = 50 µm). (**B**) Quantification of the green CellROX fluorogenic probe fluorescence intensity in *B. serrata*-treated cells compared with untreated controls (** = *p* < 0.01, *** = *p* < 0.001, n = 4). (**C**) CFU quantification of viable intracellular *P. gingivalis* treated with or without H_2_O_2_ or *B. serrata*, and pretreated or not with NAC, at 9 h post-infection. (** = *p* < 0.01, **** = *p* < 0.0001, n = 6).

To investigate the mechanism by which *B. serrata* extracts promote intracellular *P. gingivalis* clearance in oral cells, we measured ROS production, since it has been described as a microbicidal mechanism against bacterial infection. Using the cellROX fluorogenic probe, we assessed intracellular ROS production in *P. gingivalis*-infected human gingival epithelial cells in the presence or absence of *B. serrata* extracts. The fluorescence micrograph (Figure 3A) and corresponding fluorescence quantification (Figure 3B) revealed a significant increase in ROS production in *P. gingivalis*-infected cells treated with *B. serrata* compared with untreated controls. To confirm whether ROS production was associated with the decrease in bacterial load found at 7 h and 9 h post-infection (Figure 2), we performed CFU quantification of *P. gingivalis* in gingival cells pretreated with the anti-oxidant N-acetylcysteine (NAC). Our analysis demonstrated that treatment with *B. serrata* extracts and H_2_O_2_ (the positive control) reduced intracellular *P. gingivalis* survival, while pretreatment with NAC blocked *B. serrata*-induced bacterial elimination (Figure 3C). These findings suggest that *B. serrata* extracts induce elimination of intracellular *P. gingivalis* in human oral cells via ROS-mediated production.

### 2.4. 3-O-Acetyl-11-Keto-β-Boswellic Acids (AKBA), but Not β-Boswellic Acids (BA), Promote ROS-Mediated Antimicrobial Activity

**Figure 4:**
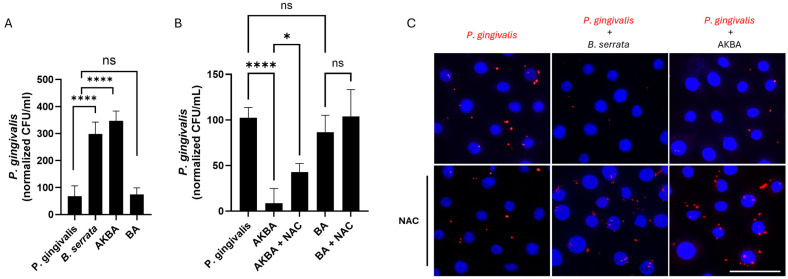
AKBA promotes the killing of *P. gingivalis* through ROS production, reproducing the *B. serrata* phenotype. (**A**) CFU quantification of viable intracellular *P. gingivalis* treated with or without *B. serrata* extract or its bioactive components AKBA or BA at 5 h post-infection (**** = *p* < 0.0001, ns = no significance, n = 6). (**B**) CFU quantification of viable intracellular *P. gingivalis* treated with or without AKBA or BA, and pretreated or not with NAC, at 9 h post-infection (* = *p* < 0.05, **** = *p* < 0.0001, ns = no significance, n = 4). (**C**) Immunofluorescence micrograph showing intracellular *P. gingivalis* (red) in human gingival epithelial cells incubated with or without *B. serrata* extracts or AKBA, and pretreated or not with NAC, after 9 h of infection. (scale bar = 50 µm).

To begin to identify the bioactive compounds involved in the antibacterial effects of whole *B. serrata* extracts, we tested two known compounds found in *B. serrata* extracts. We infected human gingival epithelial cells with *P. gingivalis* for 2 h in the presence or absence of 3-O-acetyl-11-keto-β-boswellic acid (AKBA) or β-boswellic acid (BA). At early times of infection (5 h post-infection), AKBA treatment significantly increased intracellular bacterial load to levels comparable to those observed when cells were treated with whole *B. serrata* extracts (Figure 4A). On the other hand, BA did not show any effect on *P. gingivalis* internalization in oral cells (Figure 4A).

At later times of infection (9 h post-infection), CFU quantification showed a significant decrease in *P. gingivalis* intracellular load in cells treated with AKBA (Figure 4B). Pretreatment with NAC significantly blocked the AKBA-induced antibacterial effects (Figure 4B). Additionally, treatment with BA alone did not show any effect on the survival of *P. gingivalis* in oral cells (Figure 4B). The immunofluorescence micrograph (Figure 4C) confirmed the data with CFU counts (Figure 4B), showing a reduced bacterial load in *B. serrata*- or AKBA-treated cells, whereas NAC pretreatment restored the bacterial load to the level of the untreated control (Figure 4C). These results indicate that ROS production is involved in the microbicidal effects mediated by AKBA in *P. gingivalis*-infected oral cells. Altogether, these results suggest that AKBA, but not BA, contributes to the antibacterial effects of *B. serrata* extracts against *P. gingivalis*.

## 3. Discussion

Frankincense has been used for centuries in traditional medicine across regions of Africa, India, and the Middle East, where *Boswellia* species are found [25]. Historically valued for their anti-inflammatory properties, *Boswellia* extracts have more recently gained attention in biomedical research, with accumulating evidence supporting their therapeutic potential across a range of inflammatory and neoplastic conditions [11]. Clinical and preclinical studies have reported beneficial effects of frankincense preparations in various diseases, including arthritis, inflammatory bowel disease, asthma, and brain tumors [11,26,27,28]. In addition to their anti-inflammatory activity, frankincense-derived compounds have been associated with anti-thrombotic, anti-ulcer, and anti-diabetic effects [11]. The constituents that have been studied the most for their therapeutic potential are pentacyclic triterpenic acids, β-boswellic acid (BA), acetyl-β-boswellic acid (ABA), 11-keto-β-boswellic acid (KBA), and 3-O-acetyl-11-keto-β-boswellic acid (AKBA) [25,29].

Beyond immunomodulation, *B. serrata* extracts have demonstrated antimicrobial activity against fungi as well as Gram-positive and Gram-negative bacteria, including *Staphylococcus epidermidis*, *Enterococcus faecalis*, and *Escherichia coli* [14]. Boswellic extracts have also been reported to inhibit growth of oral pathogens, with efficacy dependent on compound composition and concentration [12]. Our group has previously shown that *B. serrata* extract reduces the survival of *P. gingivalis* under planktonic conditions and inhibits *P. gingivalis* biofilm formation [13]. Antimicrobial activities have been attributed, at least in part, to terpenoid-mediated disruption of bacterial membranes, inhibition of protein synthesis, and impairment of ATP production [14,30].

**Figure 5:**
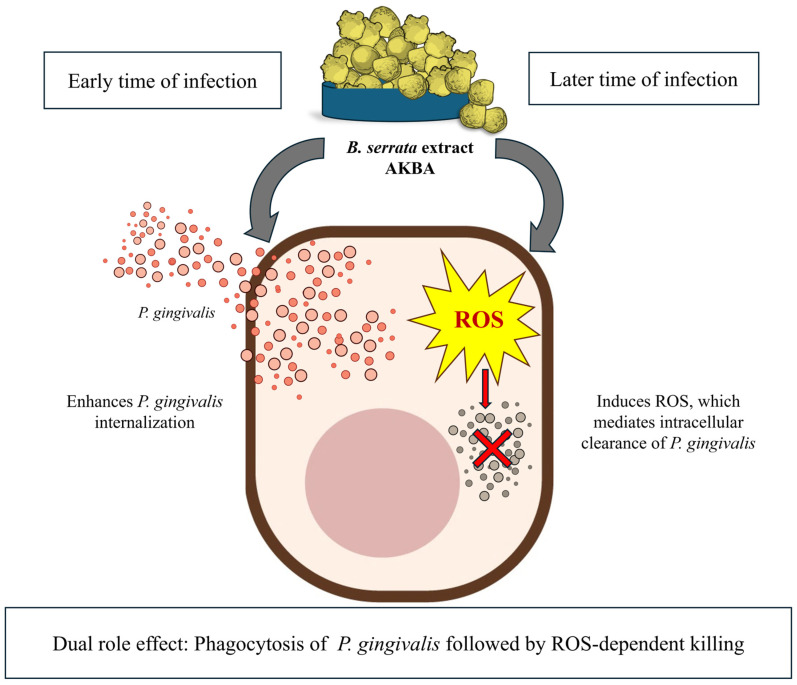
Proposed scheme of the dual antibacterial effects of *B. serrata* extract and AKBA. Schematic representation illustrating the dual role by which *Boswellia serrata* exerts antimicrobial effects against *P. gingivalis*. At early times of infection, *B. serrata* and AKBA enhance bacterial internalization by stimulating phagocytosis. At later times of infection, *B. serrata* and AKBA trigger ROS production, leading to the killing of intracellular bacteria.

Despite evidence supporting growth inhibition and direct antimicrobial effects, prior studies have not elucidated mechanisms by which *Boswellia* extracts influence host–pathogen interactions during infection. In this study, we demonstrate for the first time that whole *B. serrata* extract and its bioactive compound AKBA modulate both bacterial internalization and intracellular persistence of *P. gingivalis* in human gingival epithelial cells. Notably, treatment with *B. serrata* enhanced bacterial uptake during early times of infection, while subsequently promoting intracellular clearance at later times of infection through a ROS-dependent mechanism (Figure 5).

Similarly to other phytochemicals, such as curcumin and catechins, boswellic acids may prime epithelial cells by enhancing phagocytosis and activating pathways that precede antimicrobial responses [31,32]. Previous studies have suggested that boswellic acids regulate intracellular vesicle trafficking, glycosylation, and molecular transport, thereby influencing the function of the Golgi apparatus and autophagy-related pathways [33,34]. Our data provide the first evidence that *B. serrata* extracts directly affect the phagocytosis of both bacterial pathogens and inert particulate latex beads. Subsequent mechanisms, such as lysosomal fusion and vacuolar acidification, may also be involved in intracellular *P. gingivalis* elimination and should be further explored in future studies.

Cellular mechanisms that restrict intracellular pathogen infections are highly context-dependent and vary according to the cell type and microbial features. In experimental models using professional immune cells, pathogen clearance involves various processes, including phagocytosis, inflammasome activation, ROS and nitric oxide production, vesicular fusion, degradation within phagolysosomes, autophagy, and proteasomal pathways [35]. As part of the innate immune response, gingival epithelial cells retain the capacity to deploy antimicrobial mechanisms in response to infection [36,37].

Previous studies have shown that 11-keto-boswellic acids can stimulate ROS generation in neutrophils via NADPH oxidase and MAPK signaling [24]. In oral cancer cells, AKBA was shown to promote oxidative stress via mitochondrial ROS [21]. Conversely, in some non-immune cell types, AKBA has been reported to attenuate oxidative stress through Nrf2 activation, underscoring the context-dependent effects of boswellic acids [38,39,40,41]. In our model, oxidative stress emerged as a critical mediator of intracellular bacterial clearance, as NAC completely abrogated the antimicrobial effects of *B. serrata* extracts. Importantly, we demonstrated that AKBA was a key compound in the elimination of intracellular *P. gingivalis*, which involved ROS-dependent mechanisms, as evidenced by the ability of NAC to block the bacterial-killing effects. The primary source of ROS in our model (NADPH oxidase or mitochondrial) and other microbicidal events related to the phagocytic pathway, such as the activation of the lysosomal acidification pathway, remain to be elucidated. These findings are consistent with prior studies demonstrating the central role of ROS in reducing intracellular pathogens and the capacity of natural products to potentiate host antimicrobial responses [14,18].

As previously discussed [11], several studies have proposed using frankincense oil as a therapeutic alternative for various diseases. *B. serrata* extracts are considered safe for human consumption, and the pharmacokinetics of solid lipid *B. serrata* particles in healthy human subjects have been described for the extracts and the compounds AKBA and BA [25]. Studies have demonstrated that oral administration of *Boswellia* extracts is beneficial for the treatment of osteoarthritis [26], oral aphthous ulcers [27], and ulcerative colitis [28]. Given that previous studies have shown the efficacy of orally administered *Boswellia* extracts for other conditions, our previous study [13] and current work raises the possibility of using *Boswellia* extracts and AKBA in ointments or mouthwashes, for example, to treat periodontal disease.

Taken together, our data support a dual mechanism of action for the whole *B. serrata* extract and AKBA: an early step characterized by enhanced bacterial phagocytosis in the initial times of infection, likely reflecting epithelial activation, followed by ROS-mediated intracellular killing at later times of infection. This biphasic response suggests that *Boswellia* compounds reinforce epithelial defense mechanisms by coupling host metabolic and oxidative pathways, leading to pathogen elimination. Importantly, these findings provide new insights into how natural compounds can simultaneously modulate host immunity while limiting microbial persistence in oral epithelial infections.

From a clinical perspective, these results support the use of *B. serrata*-derived bioactive compounds as potential therapeutics in periodontal disease, where both antimicrobial activity and host modulation are desirable. Future studies should investigate additional bioactive components within frankincense extracts, explore ROS-independent mechanisms, and assess interactions with immune cells to further define the therapeutic framework for leveraging natural compounds in host-directed antimicrobial strategies.

## 4. Materials and Methods

### 4.1. Boswellia Serrata Extract and Compounds

*B. serrata* extract USP reference standard (cat #1076250, Sigma Aldrich—St. Louis, MO, USA) was resuspended in dimethyl sulfoxide (DMSO; Sigma Aldrich—St. Louis, MO, USA) and used in our experiments, as we previously described (13). Purified compounds from *B. serrata* were 3-O-Acetyl-11-keto-β-boswellic acid (AKBA—cat# PHL89169, Sigma Aldrich—St. Louis, MO, USA) and β-Boswellic acid (BA—cat # 80342, Sigma Aldrich—St. Louis, MO, USA). The compounds were resuspended in DMSO at 128mg/mL for *B. serrata*, 10 mg/mL for AKBA, and 50 mg/mL for BA. The compounds were stored at 4 °C until use.

### 4.2. Bacterial Strain and Cell Culture

*P. gingivalis* (ATCC^®^ 33277) was from American Type Culture Collection (ATTC—Manassas, Virginia, Washington, DC, USA) and grown as previously described [7,13]. Briefly, *P. gingivalis* was grown anaerobically at 37 °C for approximately 7 days in Brucella agar plates (Anaerobe systems, cat# AS-141—Morgan Hill, CA, USA). Isolated and pure colonies were collected from agar plates and inoculated into supplemented BHI broth at 37 °C under anaerobic conditions for approximately 48 h prior to the experiments. Freshly grown bacteria were used in experiments after being collected in log phase and quantified by optical density at 600 nm using the SpectraMax iD3 microplate reader (Molecular Devices—Ramsey, MN, USA).

Immortalized human gingival keratinocytes (HPV-16GM), referred to as human gingival epithelial cells, were obtained from Applied Biological Materials (ABM, cat#T0717—Richmond, CA, USA) and maintained as we previously described [7,13]. Briefly, human gingival epithelial cells were grown and maintained in keratinocyte-serum-free medium (KSFM) supplemented with 30 µg/mL of bovine pituitary extract, 0.2 ng/mL of human recombinant epidermal growth factor, 100 U/mL of penicillin, and 100 µg/mL of streptomycin (Gibco—Gaithersburg, MO, USA). The cells were grown in a humidified incubator at 37 °C, 5% CO_2_, and quantified using trypan blue (Sigma-Aldrich—St. Louis, MO, USA) exclusion before each experiment.

### 4.3. Quantification of P. gingivalis Infection

Antibiotic protection assays were performed to quantify intracellular *P. gingivalis* in human gingival epithelial cells, as we previously described [7,13]. Briefly, 3 × 10^5^ human gingival epithelial cells were seeded in 6-well plates (Costar, Corning—Glendale, AZ, USA) and incubated overnight in supplemented KSFM media without antibiotics. The cells were infected with *P. gingivalis* at an MOI of 100 in the presence or absence of *B. serrata* extracts or its compounds AKBA and BA at concentrations of 16 µg/mL, 4 µg/mL, and 32 µg/mL, respectively, in OptiMEM (Gibco, Gaithersburg, MO, USA) for 2 h, at 37 °C, 5% CO_2_. Then, the cells were washed three times with sterile prewarmed PBS. To remove extracellular bacteria, the cells were treated with metronidazole (200 µg/mL) and gentamicin (300 µg/mL) in OptiMEM for an additional 1 h at 37 °C and 5% CO_2_. Then, the cells were washed three times with prewarmed PBS and maintained in OptiMEM for up to 5 h, or for a total of 9 h. After 5 h or 9 h of infection, sterile distilled water was added to the wells and incubated at room temperature for 20 min to lyse the cells. Each cell lysate was plated onto a Brucella Blood Agar Plate (Anaerobe Systems, Morgan Hill, CA, USA) and incubated under anaerobic conditions for 10 days at 37 °C. The colony-forming units (CFU) were then counted and quantified.

For the detection of *P. gingivalis* infection using immunofluorescence microscopy, 1 × 10^5^ human gingival epithelial cells were seeded onto a 13 mm coverslip in a 24-well plate and incubated overnight at 37 °C and 5% CO_2_. The experiment followed the description above for the “colony-forming unit assays”, in which the cells were infected for 2 h in the presence or absence of *B. serrata* extract or its compounds AKBA and BA at concentrations of 16 µg/mL, 4 µg/mL, and 32 µg/mL, respectively. Then, the cells were treated with antibiotics for an additional 1 h and incubated in OptiMEM at 37 °C, 5% CO_2_ for up to 5 h, 7 h, or 9 h. In some experiments, we pretreated the cells with NAC (10 µM) 1 h before infection. At the end of the experiment, infected cells on the coverslips were washed three times with sterile PBS, then fixed with 4% formaldehyde. The cells were washed three times with PBS, permeabilized, and blocked with a solution of 0.2% Triton X-100 (Sigma Aldrich—St. Louis, MO, USA) in 5% Goat Serum/PBS (Sigma Aldrich—St. Louis, MO, USA) for 40 min, at RT. The cells were incubated with a primary rabbit polyclonal antibody anti-*P. gingivalis* (cat# ANT0085, Diatheva—Cartoceto, Italy) at a concentration of 1:50 prepared in 0.05% Triton X-100 in 5% Goat Serum/PBS at 4 °C, for 3 h. After three washes with PBS, secondary goat anti-rabbit IgG (cat# A11012, Invitrogen—Carlsbad, CA, USA) at a concentration of 1:200 was prepared in 0.05% Triton X-100 in 5% Goat Serum/PBS, and was added into the wells for incubation for 2 h, protected from light, at RT. The coverslips were washed three times and mounted onto a slide using Vectashield Hardset Antifade Mounting Medium with DAPI (Cat # H-1500, Vector Laboratories, Burlingame, CA, USA). Images were then acquired using a Nikon Eclipse 50i fluorescence microscope with an Infinity 3 camera (Nikon Instruments, Melville, NY, USA) and the Lumenera Infinity Analyze 6.3 software (Teledyne Lumenera, Ottawa, ON, Canada), or a THUNDER imager 3D assay microscope (Leica, Wetzlar, Germany). Fluorescence intensity was quantified using the ImageJ software (version 1.54x, National Institutes of Health, Bethesda, MD, USA). The background fluorescence was obtained from adjacent cell-free regions and subtracted from all measurements. Data represent the mean fluorescence intensity expressed as arbitrary units.

### 4.4. Phagocytosis Assay

For the detection of phagocytosis using fluorescence microscopy, 1 × 10^5^ human gingival epithelial cells were seeded overnight onto a 13 mm coverslip in a 24-well plate at 37 °C and 5% CO_2_. The cells were incubated with 0.5 μm or 1 μm green fluorescent latex beads (Cat # F-7895, Thermo Fisher Scientific, Waltham, MA, USA) for 2 h at a cell/bead ratio of 1:100 in the presence or absence of 16 µg/mL of *B. serrata* extracts. Then, the cells were washed three times with sterile PBS and incubated in OptiMEM for an additional 3 h at 37 °C and 5% CO_2_, for a total of 5 h. The cells were fixed with 4% formaldehyde solution for 30 min. The coverslips were washed three times and mounted onto a slide using Vectashield Hardset Antifade Mounting Medium with DAPI (Cat # H-1500, Vector Laboratories, Burlingame, CA, USA) and Vectashield Hardset Antifade Mounting Medium with phalloidin (Cat # H-1600, Vector Laboratories, Burlingame, CA, USA). Images were then acquired using a Nikon Eclipse 50i fluorescence microscope with an Infinity 3 camera (Nikon Instruments, Melville, NY, USA) and the Lumenera Infinity Analyze 6.3 software (Teledyne Lumenera, Ottawa, ON, Canada), or a THUNDER imager 3D assay microscope (Leica). Using ImageJ, the percentage of bead-containing cells was calculated as (nBCC × 100)/total C, where nBCC is the number of bead-containing cells, and total C is the total number of cells. The results are expressed as a percentage (%) of bead-positive cells.

### 4.5. Measurement of ROS Production

For detection of ROS production using fluorescence microscopy, 1 × 10^5^ human gingival epithelial cells were seeded overnight onto a 13 mm coverslip in a 24-well plate at 37 °C and 5% CO_2_. The experiment followed the description above for the “colony-forming units assay”, in which the cells were infected for 2 h in the presence or absence of *B. serrata* extracts, treated with antibiotics for an additional 1 h, and then incubated in OptiMEM for an additional 4 h, at 37 °C, 5% CO_2_ for a total of 7 h of infection. CellROX green fluorogenic probe was added to the wells 1 h before the end of the experiment. Hydrogen peroxide was used as a positive control. At the end of the experiment, the cells on the coverslips were washed three times with sterile PBS, then fixed with 4% formaldehyde for 30 min. The coverslips were washed three times and mounted onto a slide using Vectashield Hardset Antifade Mounting Medium with DAPI (Cat # H-1500, Vector Laboratories, Burlingame, CA, USA). Images were then acquired using a Nikon Eclipse 50i fluorescence microscope with an Infinity 3 camera (Nikon Instruments, Melville, NY, USA) and the Lumenera Infinity Analyze 6.3 software (Teledyne Lumenera, Ottawa, ON, Canada), or a THUNDER imager 3D assay microscope (Leica). The fluorescence intensity was quantified using ImageJ software and expressed as arbitrary units.

### 4.6. Statistics

Statistical analysis was performed using Prism GraphPad (GraphPad Software, version 9.5.1). The results are presented as standard deviation (SD) and were analyzed using an unpaired *t*-test with Welch’s correction for up to two conditions and one-way ANOVA followed by Tukey’s multiple comparison test for more than two analyzed conditions. Differences resulting in *p* < 0.05 were considered significant.

## 5. Conclusions

*B. serrata* extracts and AKBA exhibit antimicrobial activity against *P. gingivalis*, which is mediated in part by the enhancement of host ROS-dependent clearance mechanisms. These findings highlight AKBA as a promising compound for the development of therapeutics derived from *B. serrata*. More broadly, this study reinforces the relevance of plant-derived compounds as modulators of host–pathogen interactions, thereby expanding their potential applications in oral health and the management of infectious diseases.

## Data Availability

The original contributions presented in this study are included in the article. Further inquiries can be directed to the corresponding author(s).

## References

[1] Lee K., Roberts J.S., Choi C.H., Atanasova K.R., Yilmaz O. (2018). *Porphyromonas gingivalis* traffics into endoplasmic reticulum-rich-autophagosomes for successful survival in human gingival epithelial cells. Virulence.

[2] Yilmaz O., Jungas T., Verbeke P., Ojcius D.M. (2004). Activation of the phosphatidylinositol 3-kinase/Akt pathway contributes to survival of primary epithelial cells infected with the periodontal pathogen *Porphyromonas gingivalis*. Infect. Immun..

[3] Yilmaz O., Verbeke P., Lamont R.J., Ojcius D.M. (2006). Intercellular spreading of *Porphyromonas gingivalis* infection in primary gingival epithelial cells. Infect. Immun..

[4] Bui F.Q., Almeida-da-Silva C.L.C., Huynh B., Trinh A., Liu J., Woodward J., Asadi H., Ojcius D.M. (2019). Association between periodontal pathogens and systemic disease. Biomed. J..

[5] Hajishengallis G. (2015). Periodontitis: From microbial immune subversion to systemic inflammation. Nat. Rev. Immunol..

[6] Makkawi H., Hoch S., Burns E., Hosur K., Hajishengallis G., Kirschning C.J., Nussbaum G. (2017). *Porphyromonas gingivalis* Stimulates TLR2-PI3K Signaling to Escape Immune Clearance and Induce Bone Resorption Independently of MyD88. Front. Cell. Infect. Microbiol..

[7] Almeida-da-Silva C.L.C., Ramos-Junior E.S., Morandini A.C., Rocha G.D.C., Marinho Y., Tamura A.S., de Andrade K.Q., Bellio M., Savio L.E.B., Scharfstein J. (2019). P2X_7_ receptor-mediated leukocyte recruitment and *Porphyromonas gingivalis* clearance requires IL-1β production and autocrine IL-1 receptor activation. Immunobiology.

[8] Ramos-Junior E.S., Morandini A.C., Almeida-da-Silva C.L.C., Franco E.J., Potempa J., Nguyen K.A., Oliveira A., Zamboni D., Ojcius D., Scharfstein J. (2015). A Dual Role for P2X_7_ Receptor during *Porphyromonas gingivalis* Infection. J. Dent. Res..

[9] Morandini A.C., Ramos-Junior E.S., Potempa J., Nguyen K.A., Oliveira A.C., Bellio M., Ojcius D.M., Scharfstein J., Coutinho-Silva R. (2014). *Porphyromonas gingivalis* fimbriae dampen P2X_7_-dependent IL-1β secretion. J. Innate Immun..

[10] Miethke M., Pieroni M., Weber T., Bronstrup M., Hammann P., Halby L., Arimondo P.B., Glaser P., Aigle B., Bode H.B. (2021). Towards the sustainable discovery and development of new antibiotics. Nat. Rev. Chem..

[11] Almeida-da-Silva C.L.C., Sivakumar N., Asadi H., Chang-Chien A., Qoronfleh M.W., Ojcius D.M., Essa M.M. (2022). Effects of Frankincense Compounds on Infection, Inflammation, and Oral Health. Molecules.

[12] Raja A.F., Ali F., Khan I.A., Shawl A.S., Arora D.S. (2011). Acetyl-11-keto-β-boswellic acid (AKBA); targeting oral cavity pathogens. BMC Res. Notes.

[13] Vang D., Moreira-Souza A.C.A., Zusman N., Moncada G., Matshik Dakafay H., Asadi H., Ojcius D.M., Almeida-Da-Silva C.L.C. (2024). Frankincense (*Boswellia serrata*) Extract Effects on Growth and Biofilm Formation of *Porphyromonas gingivalis*, and Its Intracellular Infection in Human Gingival Epithelial Cells. Curr. Issues Mol. Biol..

[14] Jaros P., Timkina E., Michailidu J., Marsik D., Kulisova M., Kolouchova I., Demnerová K. (2022). Boswellic Acids as Effective Antibacterial Antibiofilm Agents. Molecules.

[15] Choi C.H., Spooner R., DeGuzman J., Koutouzis T., Ojcius D.M., Yilmaz O. (2013). *Porphyromonas gingivalis*-nucleoside-diphosphate-kinase inhibits ATP-induced reactive-oxygen-species via P2X_7_ receptor/NADPH-oxidase signalling and contributes to persistence. Cell. Microbiol..

[16] Hung S.C., Choi C.H., Said-Sadier N., Johnson L., Atanasova K.R., Sellami H., Yilmaz Ö., Ojcius D.M. (2013). P2X_4_ assembles with P2X_7_ and pannexin-1 in gingival epithelial cells and modulates ATP-induced reactive oxygen species production and inflammasome activation. PLoS ONE.

[17] Nathan C., Cunningham-Bussel A. (2013). Beyond oxidative stress: An immunologist’s guide to reactive oxygen species. Nat. Rev. Immunol..

[18] Alfei S., Schito G.C., Schito A.M., Zuccari G. (2024). Reactive Oxygen Species (ROS)-Mediated Antibacterial Oxidative Therapies: Available Methods to Generate ROS and a Novel Option Proposal. Int. J. Mol. Sci..

[19] Moreira-Souza A.C.A., Almeida-da-Silva C.L.C., Rangel T.P., Rocha G.D.C., Bellio M., Zamboni D.S., Vommaro R.C., Coutinho-Silva R. (2017). The P2X_7_ Receptor Mediates Toxoplasma gondii Control in Macrophages through Canonical NLRP3 Inflammasome Activation and Reactive Oxygen Species Production. Front. Immunol..

[20] Ahmed S.A., Al-Shanon A.F., Al-Saffar A.Z., Tawang A., Al-Obaidi J.R. (2023). Antiproliferative and cell cycle arrest potentials of 3-O-acetyl-11-keto-β-boswellic acid against MCF-7 cells in vitro. J. Genet. Eng. Biotechnol..

[21] Li C., He Q., Xu Y., Lou H., Fan P. (2022). Synthesis of 3-O-Acetyl-11-keto-β-boswellic Acid (AKBA)-Derived Amides and Their Mitochondria-Targeted Antitumor Activities. ACS Omega.

[22] Mohamed H.R.H., Ibrahim E.G.S., El-Sherif A.A. (2025). Frankincense oil nanoemulsion induces selective cytotoxicity and over ROS-mediated oxidative stress and apoptotic DNA damage in Hep-G2 hepatic cancer cells. Sci. Rep..

[23] Pan D., Wang Q., Tang S., Wu X., Cai L., Wang Z., Li Y., Huang M., Zhou Y., Shen Y.-Q. (2024). Acetyl-11-keto-β-boswellic acid inhibits cell proliferation and growth of oral squamous cell carcinoma via RAB7B-mediated autophagy. Toxicol. Appl. Pharmacol..

[24] Altmann A., Poeckel D., Fischer L., Schubert-Zsilavecz M., Steinhilber D., Werz O. (2004). Coupling of boswellic acid-induced Ca^2+^ mobilisation and MAPK activation to lipid metabolism and peroxide formation in human leucocytes. Br. J. Pharmacol..

[25] Siddiqui M.Z. (2011). *Boswellia serrata*, a potential antiinflammatory agent: An overview. Indian J. Pharm. Sci..

[26] Krieglstein C.F., Anthoni C., Rijcken E.J., Laukotter M., Spiegel H.U., Boden S.E., Schweizer S., Safayhi H., Senninger N., Schürmann G. (2001). Acetyl-11-keto-β-boswellic acid, a constituent of a herbal medicine from *Boswellia serrata* resin, attenuates experimental ileitis. Int. J. Color. Dis..

[27] Gerhardt H., Seifert F., Buvari P., Vogelsang H., Repges R. (2001). Therapy of active Crohn disease with *Boswellia serrata* extract H 15. Z. Gastroenterol..

[28] Majeed M., Majeed S., Narayanan N.K., Nagabhushanam K. (2019). A pilot, randomized, double-blind, placebo-controlled trial to assess the safety and efficacy of a novel *Boswellia serrata* extract in the management of osteoarthritis of the knee. Phytother. Res..

[29] Cherepanova M.O., Subotyalov M.A. (2023). Component Composition and Biological Activity of Oleo-Gum Resin from *Boswellia serrata* (Burseraceae). Dokl. Biol. Sci..

[30] Raja A.F., Ali F., Khan I.A., Shawl A.S., Arora D.S., Shah B.A., Taneja S.C. (2011). Antistaphylococcal and biofilm inhibitory activities of acetyl-11-keto-β-boswellic acid from *Boswellia serrata*. BMC Microbiol..

[31] Cho J.A., Park E. (2015). Curcumin utilizes the anti-inflammatory response pathway to protect the intestine against bacterial invasion. Nutr. Res. Pract..

[32] Daglia M. (2012). Polyphenols as antimicrobial agents. Curr. Opin. Biotechnol..

[33] Yang Y.H., Li W., Ren L.W., Yang H., Zhang Y.Z., Zhang S., Hao Y., Yu D.-K., Tong R.-S., Du G.-H. (2024). S670, an amide derivative of 3-O-acetyl-11-keto-β-boswellic acid, induces ferroptosis in human glioblastoma cells by generating ROS and inhibiting STX17-mediated fusion of autophagosome and lysosome. Acta Pharmacol. Sin..

[34] Nakano K., Sasaki S., Kataoka T. (2022). Bioactive Evaluation of Ursane-Type Pentacyclic Triterpenoids: β-Boswellic Acid Interferes with the Glycosylation and Transport of Intercellular Adhesion Molecule-1 in Human Lung Adenocarcinoma A549 Cells. Molecules.

[35] Diacovich L., Gorvel J.P. (2010). Bacterial manipulation of innate immunity to promote infection. Nat. Rev. Microbiol..

[36] Schleimer R.P., Kato A., Kern R., Kuperman D., Avila P.C. (2007). Epithelium: At the interface of innate and adaptive immune responses. J. Allergy Clin. Immunol..

[37] Santana P.T., Martel J., Lai H.C., Perfettini J.L., Kanellopoulos J.M., Young J.D., Coutinho-Silva R., Ojcius D.M. (2016). Is the inflammasome relevant for epithelial cell function?. Microbes Infect..

[38] Ammon H.P. (2016). Boswellic Acids and Their Role in Chronic Inflammatory Diseases. Adv. Exp. Med. Biol..

[39] Fakhri K.U., Sharma D., Fatma H., Yasin D., Alam M., Sami N., Ahmad F.J., Shamsi A., Alam Rizvi M. (2025). The Dual Role of Dietary Phytochemicals in Oxidative Stress: Implications for Oncogenesis, Cancer Chemoprevention, and ncRNA Regulation. Antioxidants.

[40] Zhou C., Wang Y., Zhang Q., Zhou G., Ma X., Jiang X., Yu W. (2023). Acetyl-11-Keto-β-Boswellic Acid Activates the Nrf2/HO-1 Signaling Pathway in Schwann Cells to Reduce Oxidative Stress and Promote Sciatic Nerve Injury Repair. Planta Med..

[41] Ahmad S., Khan S.A., Kindelin A., Mohseni T., Bhatia K., Hoda M.N., Ducruet A.F. (2019). Acetyl-11-keto-β-boswellic acid (AKBA) Attenuates Oxidative Stress, Inflammation, Complement Activation and Cell Death in Brain Endothelial Cells Following OGD/Reperfusion. Neuromol. Med..

